# Impact of chronic constipation symptoms on work productivity and daily activity: A large‐scale internet survey

**DOI:** 10.1002/jgh3.70042

**Published:** 2024-11-04

**Authors:** Takumi Ota, Shinji Kuratani, Hisanori Masaki, Sonoko Ishizaki, Haruhiko Seki, Takahiro Takebe

**Affiliations:** ^1^ Medical Affairs Department Mochida Pharmaceutical Co., Ltd. Shinjuku‐ku Tokyo Japan; ^2^ Medical Science Group, Medical Department EA Pharma Co., Ltd. Chuo‐ku Tokyo Japan; ^3^ Value and Access Department INTAGE Healthcare Inc. Chiyoda‐ku Tokyo Japan

**Keywords:** activity impairment, chronic constipation, daily activity, work productivity, WPAI‐CC

## Abstract

**Background and Aim:**

Chronic constipation negatively impacts work productivity and patients' quality of life. This retrospective study assessed the correlation between symptoms of chronic constipation and work/activity impairment with and without the use of laxative treatment.

**Methods:**

This cross‐sectional, observational, web‐based survey was conducted using the Work Productivity and Activity Impairment‐Chronic Constipation Questionnaire and included Japanese patients with chronic constipation receiving prescribed medication. Outcomes of interest included total work productivity and activity impairment and their correlation with constipation symptoms.

**Results:**

Among the 2351 analyzed patients (mean [SD] age, 51.7 [13.8] years), 80.7% were females, 63.3% had a disease duration of ≥10 years, and 1424 were working. The averages of total activity impairment, total work productivity impairment, presenteeism, and absenteeism were 39.2%, 33.9%, 31.2%, and 5.0%, respectively. The annual work productivity loss per patient was estimated to be 1.343 million Japanese Yen. Symptoms that had a statistically significant positive correlation with total work impairment (*P* < 0.05) were abdominal discomfort/nausea, abdominal pain, abdominal bloating, and unpredictable defecation timing. Total activity impairment was significantly (*P* < 0.05) affected by abdominal discomfort/nausea, abdominal bloating, abdominal pain, incomplete defecation, unpredictable defecation timing, loss of defecation desire, and straining. Work productivity and daily activity had improved in 71.2% and 72.6% of patients, respectively, after they received treatment.

**Conclusion:**

Symptoms of constipation, particularly abdominal symptoms and unpredictable defecation timing, can have a negative impact on work productivity and daily activity. Treatment focused on these symptoms may reduce the socio‐economic burden of chronic constipation in Japan.

## Graphical abstract

This retrospective, cross‐sectional, observational, web‐based survey assessed the correlation between symptoms of chronic constipation and work/activity impairment among 2351 patients. The averages of total activity impairment, total work productivity impairment, presenteeism, and absenteeism due to chronic constipation were 39.2%, 33.9%, 31.2%, and 5.0%, respectively. Multivariate linear regression analysis showed a significant (*P* < 0.05) impact of chronic constipation symptoms, such as abdominal discomfort/nausea, abdominal pain, abdominal bloating, unpredictable defecation timing, and infrequent defecation, on total work impairment.

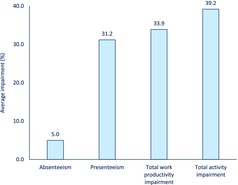

## Introduction

Chronic constipation affects up to 20% of the population globally and has a substantially negative impact on the quality of life (QoL) of patients.^1^ Even when uniform symptom‐based criteria are used for diagnosis, significant heterogeneity in prevalence is observed across countries.^2^ Symptoms of constipation can vary across individual patients and include infrequent laxation, lumpy/hard stools, degree of straining, sensation of incomplete evacuation, loss of urge of defecation, etc.^3^, ^4^, ^5^

According to the Evidence‐Based Clinical Practice Guideline for Chronic Constipation 2023, constipation means “a condition in which a stool that should be evacuated is retained in the colon, resulting in fecal impaction and hard stools, and decreased frequency of bowel movements and/or excessive irritation, residual stool sensation, obstruction of the rectum and difficulty in defecation due to inability to evacuate stool comfortably.” The guideline also defines chronic constipation as “a condition in which patients suffer from chronic constipation that interferes with their daily life and/or may cause various physical problems.”^6^ In addition to this, Rome IV, the internationally recognized diagnostic criteria for functional gastrointestinal diseases, defines functional constipation as a condition that meets two or more of the six prespecified conditions: straining, lumpy/hard stools, sensation of incomplete evacuation, sensation of anorectal obstruction/blockage, manual maneuvers to facilitate defecation, and decreased frequency of stools. Rome IV recommends that these conditions should be a part of all diagnostic criteria utilized for constipation assessment.^7^

Initial management of constipation relies on lifestyle modifications and the use of non‐pharmacologic agents.^1^ However, when these strategies show suboptimal improvement, the use of pharmacologic agents is employed. The treatment algorithm for chronic constipation includes the use of various bulk laxatives, stimulant laxatives, stool softeners, prokinetic agents, and drug therapies.^1^ Until a few years ago, the mainstays of treatment for chronic constipation in Japan were osmotic laxatives such as magnesium oxide and irritant laxatives of the anthraquinone and diphenyl groups.^8^ In spite of the various therapy options, many patients remain dissatisfied with treatment due to the varied and complex symptoms of chronic constipation. Therefore, new agents such as lubiprostone, linaclotide, and elobixibat have been developed and become available for constipation therapy.^8^, ^9^

Chronic constipation symptoms are often severe and bothersome, with a negative impact on patients' QoL,^10^ thus affecting their work productivity and other daily activities.^11^ This loss of productivity is estimated to be directly proportional to the severity of patients' symptoms.^12^ It has been reported that the health burden associated with chronic constipation can be substantially higher than that experienced by patients with type 2 diabetes or reflux esophagitis and, in some cases, may be equated to the experience of those affected by irritable bowel syndrome (IBS)^13^ or chronic allergies.^14^ While it has been well established that chronic constipation leads to lower work productivity and impacts other daily activities, there is limited evidence on which symptoms of chronic constipation are specifically associated with these outcomes.

The objective of this study was to carry out exploratory research on the correlation of chronic constipation symptoms with lowering of work productivity and other daily activities and understand the impact of treatment on these outcomes in patients receiving treatment for constipation. Identification of specific symptoms that have the maximum impact on productivity loss will provide valuable information for developing treatment strategies for these patients.

## Methods

### Study design

This was a cross‐sectional, observational study utilizing a web‐based survey to assess Japanese patients with chronic constipation who were being treated with prescribed medication. The survey was conducted among patients registered with the Rakuten Insight Online Panel between 18 July 2023 and 2 August 2023.

### Study patients

Patients with chronic constipation who were using prescribed medication at the time of participation in the survey, registered in the Rakuten Insight Disease Panel or the Rakuten Insight Online Panel (Rakuten Insight, Inc., Tokyo, Japan), and ready to provide consent for participation, were included in this survey. Working patients were defined as those who answered “yes” to the question “Are you currently working in a job with monetary compensation?” Patients who were working in advertising, broadcasting, research, consulting, and pharmaceutical and medical fields were thought to be familiar with marketing research or survey responses and were excluded from the study to avoid risk of biased responses. The target sample size was 2000 patients, with at least 1000 of them being in the working category. Since there are limited studies reporting the correlation between symptoms of chronic constipation and their potential impact on work productivity and other daily activities among patients with chronic constipation, no formal a priori calculation of the sample size for the study was carried out.

### Data collection and management

The survey was conducted using a validated and commonly used Work Productivity and Activity Impairment‐Chronic Constipation (WPAI‐CC) Questionnaire written in Japanese.^12^ The detailed questionnaire is provided in Table S1, Supporting information. This work productivity rating scale is used to measure the impact of chronic constipation on patients' work productivity and the valuation of lost productivity. It consists of five items: hours lost due to constipation‐related health problems (Q2), hours lost due to any other reason (Q3), hours actually worked (Q4), degree to which constipation‐related health problems affected productivity while at work (Q5), and degree to which constipation‐related health problems affected daily activities (Q6) (excluding work). The questionnaire also collected patients' background information such as age, sex, employment status, the presence/absence of other comorbid conditions, and constipation symptoms.

An email containing a link to the online survey was sent to each potential patient, and electronically gathered patient responses were stored in a database. The estimated response time was around 10 min. In case of incomprehensible or inappropriate free‐text response or if the response to a complication‐related question reported a high number of complications that seemed doubtful or unreliable, the researcher excluded those responses.

### Outcomes

The work productivity outcomes included ratio of lost working hours, work impairment ratio and daily activity impairment ratio, correlation between total work impairment ratio or daily activity impairment ratio and each constipation symptom, work productivity outcomes with or without each constipation symptom (ratio of lost work hours, work impairment ratio, daily activity impairment ratio), and changes in degrees of work productivity loss or activity loss from before treatment. Further, the total work productivity loss was calculated by the following formulae^12^: absenteeism = [Q2/(Q2 + Q4)] × 100, presenteeism = Q5 × 10, total work productivity loss = [Q2/(Q2 + Q4)] + [(1 − Q2/(Q2 + Q4)) × (Q5/10)] × 100, activity impairment = Q6 × 10. Indirect costs were defined as the monetary value of total work productivity loss^15^ and calculated by using the estimated overall wages or salaries in Japanese Yen for each patient from the Ministry of Health, Labour and Welfare.^16^

### Statistical analysis

For categorical variables, number and percentages were calculated, whereas for continuous variables, mean, SD, and 95% confidence intervals (CIs) were calculated. To identify symptoms that impact work/activity impairment, univariate and multivariate analyses were carried out using work/activity impairment as the dependent variable and each constipation symptom as explanatory variables. Moreover, the *t* test was performed between groups with and without symptoms to determine their impact on the total work impairment in patients. All data were analyzed using SAS version 9.4 (SAS Institute, Cary, NC, USA).

The study was registered in the University Hospital Medical Information Network Clinical Trials Registry (UMIN000051507) and approved by the Ethics Committee of the Medical Corporation TOUKEIKAI Kitamachi Clinic Ethical Review Board (approval number: KXJ09514). All procedures were conducted in accordance with the Declaration of Helsinki and the Japanese Ethical Guidelines for Medical and Health Research Involving Human Subjects and its guidance document.

## Results

### Patient selection

**Figure 1:**
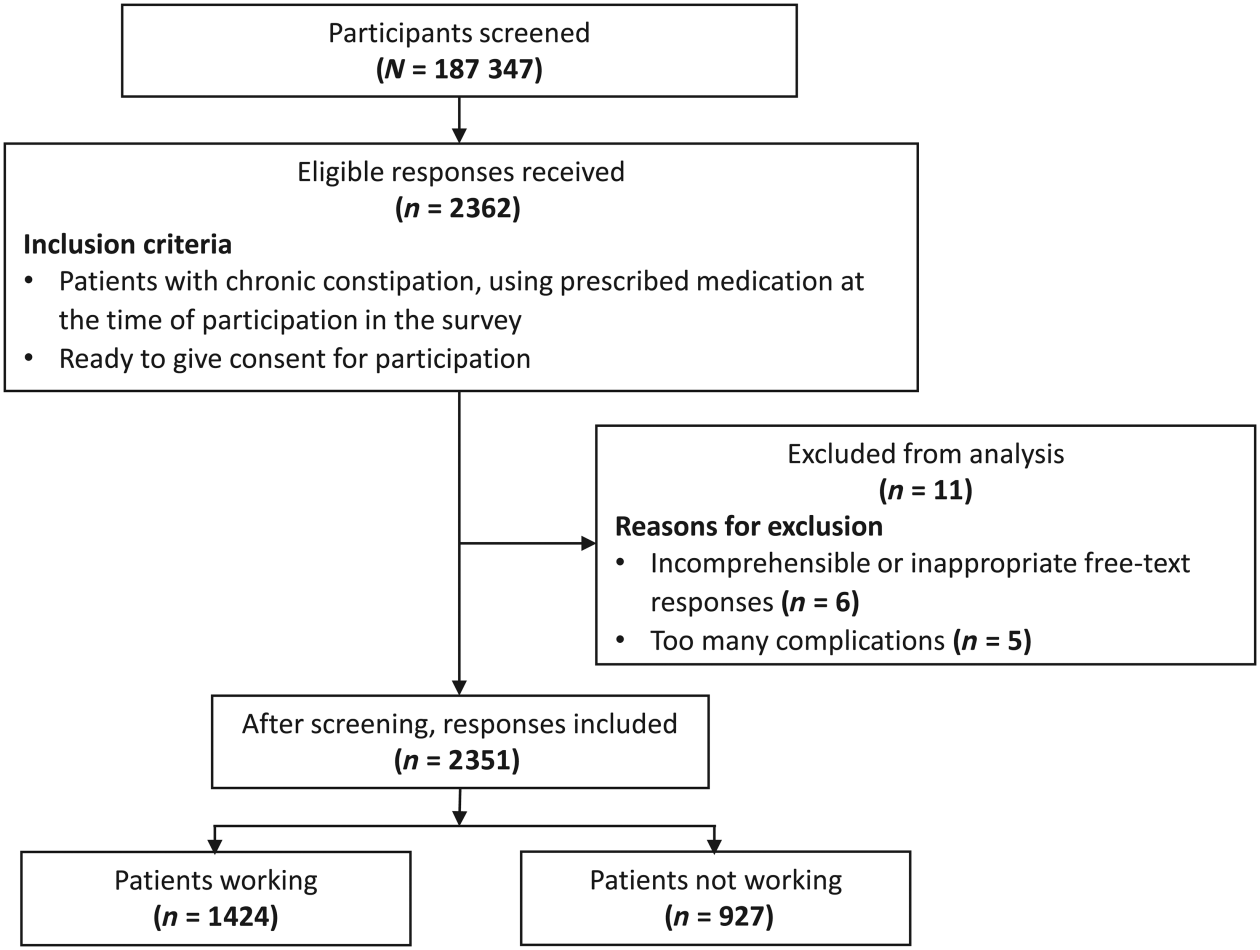
Patient flowchart.

A screening survey was conducted among a total of 187 347 participants. It identified 2362 patients with chronic constipation meeting the study's eligibility criteria. Subsequently, sample exclusion was carried out in accordance with the predefined data cleaning standards. As a result, six patients were excluded since they had incomprehensible or inappropriate free‐text response, and five patients were excluded as they reported too many complications, which made the reliability of their responses questionable. Therefore, 2351 patients were included in the analysis. A total of 1424 (60.6%) patients were working and 927 (39.4%) were not working (Fig. 1).

### Patient characteristics

The overall mean (SD) age of patients was 51.7 (13.8) years, and majority of them were female (80.7%) (Table 1). The disease duration was 10 years or longer for 63.3% of patients. A total of 42.7% of patients had no comorbid conditions. The mean (SD) number of comorbid conditions was 1.0 (1.2): hypertension, dyslipidemia, mental illness, and gastroesophageal reflux disease were the most common (approximately 19%), followed by diabetes (8.6%) and IBS (7.1%). In addition, the mean (SD) number of prescribed laxatives was 1.6 (1.0). Of these, the most prescribed laxative was magnesium oxide (70.1%), followed by stimulant laxatives (23.6%) and Chinese herbal medicine (23.4%). In about 6–8% of patients, other laxatives such as macrogol plus electrolytes, elobixibat, linaclotide, lubiprostone, and lactulose were also prescribed. Almost 70% of patients were satisfied with their current treatment.

**Table 1:** Patient characteristics

Items	Differentiation	Number of patients, *n* (%)
Age (years), mean ± SD		51.7 ± 13.8
Sex	Female	1897 (80.7)
	Male	454 (19.3)
Disease duration	<5 years	340 (14.5)
	5–10 years	393 (16.7)
	>10 years	1488 (63.3)
	Don't remember	130 (5.5)
Comorbid conditions	Hypertension	457 (19.4)
	Dyslipidemia	450 (19.1)
	Mental illness	448 (19.1)
	GERD	440 (18.7)
	Diabetes	203 (8.6)
	IBS	168 (7.1)
	Gout/hyperuricemia	85 (3.6)
	Cerebrovascular disease	65 (2.8)
	Chronic kidney disease	31 (1.3)
	Parkinson's disease	13 (0.6)
Medication prescribed for chronic constipation	Magnesium oxide	1649 (70.1)
	Stimulant laxative	556 (23.6)
	Chinese herbal medicine	550 (23.4)
	Macrogol plus electrolytes	183 (7.8)
	Elobixibat	163 (6.9)
	Linaclotide	141 (6.0)
	Lubiprostone	139 (5.9)
	Enema	98 (4.2)
	Suppositories	88 (3.7)
	Lactulose	41 (1.7)
	Other	70 (3.0)

Data are presented as *n* (%) unless specified.

GERD, gastroesophageal reflux disease; IBS, irritable bowel syndrome.

### Percentage of each constipation symptom

**Figure 2:**
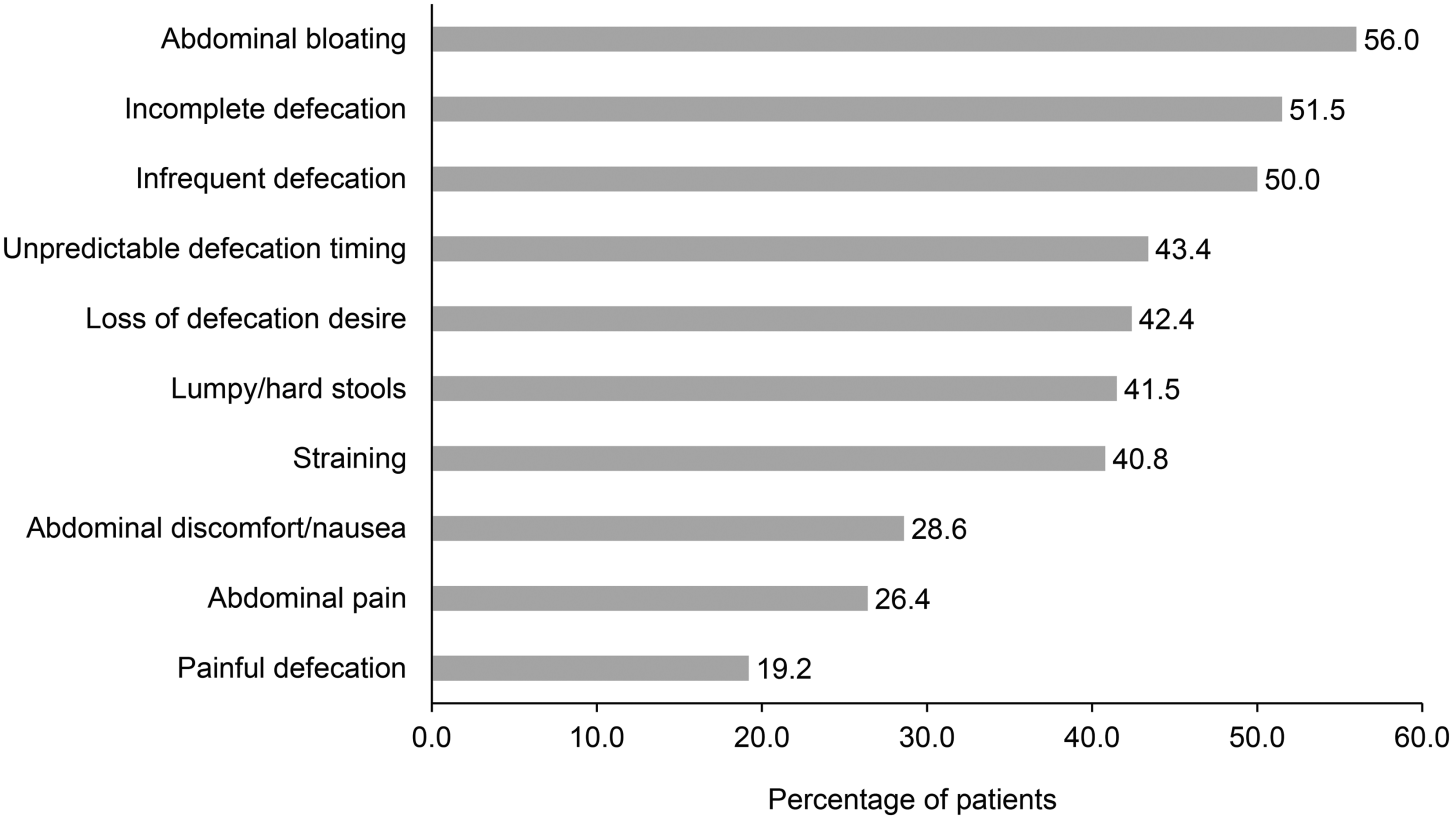
Percentage of patients with each symptom.

On average (mean [SD]), 4.0 (2.2) symptoms of chronic constipation were present in the patients. The most common symptoms of chronic constipation reported by 40–55% of patients were abdominal bloating, incomplete defecation, infrequent defecation, unpredictable defecation timing, loss of defecation desire, lumpy/hard stools, and straining. Symptoms such as abdominal discomfort/nausea, abdominal pain, and painful defecation were also reported by 20–30% of patients (Fig. 2). Further, the average number of symptoms were similar between working (mean [SD]: 4.1 [2.2]) and non‐working (mean [SD]: 3.8 [2.2]) patients.

### Total work productivity impairment, total activity impairment, and work productivity loss

**Figure 3:**
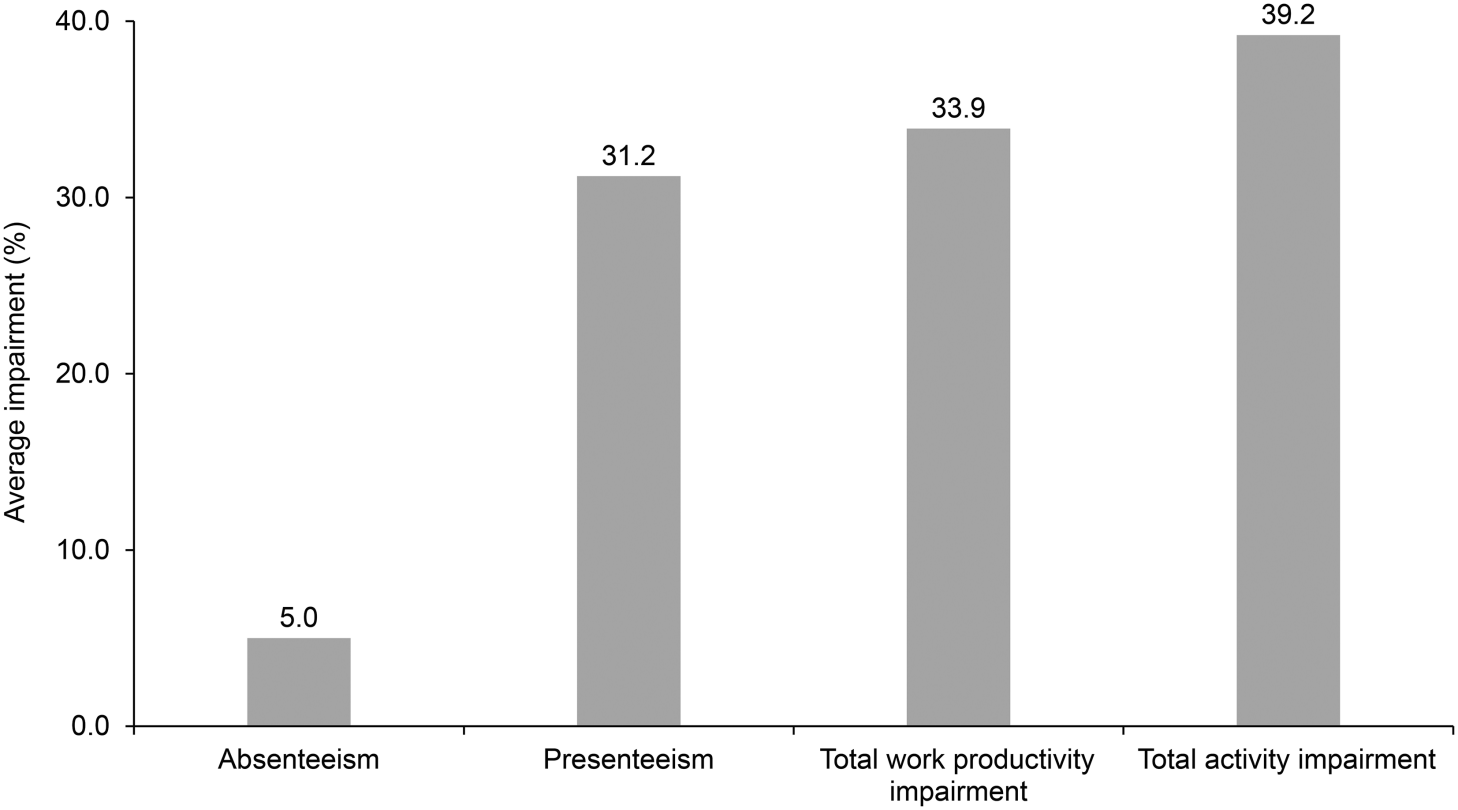
Total work productivity or activity impairment in patients with chronic constipation.

The mean (SD) total activity impairment was 39.2% (29.0%), total work productivity impairment was 33.9% (30.1%), and presenteeism was 31.2% (28.3%), whereas absenteeism due to chronic constipation was 5.0% (15.7%) (Fig. 3). The annual work productivity loss (calculated from total work impairment) per patient due to chronic constipation was 1.343 million Japanese Yen.

### Correlation between total work productivity impairment, total activity impairment, and constipation symptoms

Results of the univariate and multivariate analyses carried out for identifying symptoms that impacted total work productivity or total activity are shown in Tables 2 and 3. Multivariate linear regression analysis showed that abdominal discomfort/nausea, abdominal pain, abdominal bloating, and unpredictable defecation timing had a statistically significant (*P* < 0.05) positive correlation with total work impairment (Table 2). Likewise, the symptoms that had a statistically significant positive correlation with activity impairment (*P* < 0.05) were abdominal discomfort/nausea, abdominal pain, abdominal bloating, unpredictable defecation timing, loss of defecation desire, incomplete defecation, and straining (Table 3).

**Table 2:** Correlations between total work impairment and each constipation symptom

Factors	Number of patients^†^, *N* (%)	Univariate regression analysis		Multivariate regression analysis	
		Regression coefficient (95% CI)	*P*‐value	Regression coefficient (95% CI)	*P*‐value
Abdominal discomfort/nausea	399 (31.1)	18.4 (15.0–21.8)	<0.001	12.1 (8.4–15.8)	<0.001
Abdominal pain	375 (29.2)	16.6 (13.1–20.2)	<0.001	11.5 (7.9–15.2)	<0.001
Abdominal bloating	766 (59.7)	13.0 (9.7–16.2)	<0.001	7.0 (3.6–10.4)	<0.001
Unpredictable defecation timing	582 (45.3)	7.2 (3.9–10.5)	<0.001	4.7 (1.5–7.9)	<0.01
Loss of defecation desire	550 (42.8)	2.3 (−1.1 to 5.6)	0.180	2.6 (−0.6 to 5.8)	0.109
Incomplete defecation	683 (53.2)	7.0 (3.7–10.3)	<0.001	1.8 (−1.5 to 5.0)	0.286
Painful defecation	267 (20.8)	6.6 (2.5–10.6)	<0.01	1.0 (−3.1 to 5.0)	0.640
Straining	526 (41.0)	3.8 (0.4–7.1)	<0.05	0.0 (−3.4 to 3.4)	0.982
Lumpy/hard stools	548 (42.7)	−0.5 (−3.9 to 2.8)	0.755	−2.8 (−6.1 to 0.6)	0.102
Infrequent defecation	682 (53.1)	−0.9 (−4.2 to 2.4)	0.587	−3.7 (−6.9 to 0.4)	<0.05

^†^ The actual number of patients included in the analysis was 1284, as 140 patients responded 0 for both Questions 2 and 4.

CI, confidence interval.

**Table 3:** Correlations between activity impairment and each constipation symptom

Factors	Number of patients, *N* (%)	Univariate regression analysis		Multivariate regression analysis	
		Regression coefficient (95% CI)	*P*‐value	Regression coefficient (95% CI)	*P*‐value
Abdominal discomfort/nausea	673 (28.6)	19.4 (16.9–21.9)	<0.001	11.2 (8.5–13.8)	<0.001
Abdominal bloating	1317 (56.0)	16.0 (13.8–18.3)	<0.001	9.2 (6.9–11.6)	<0.001
Abdominal pain	621 (26.4)	14.9 (12.3–17.5)	<0.001	8.1 (5.6–10.7)	<0.001
Incomplete defecation	1211 (51.5)	12.2 (9.9–14.5)	<0.001	6.6 (4.3–8.8)	<0.001
Unpredictable defecation timings	1021 (43.4)	7.9 (5.5–10.2)	<0.001	3.7 (1.5–5.9)	<0.01
Loss of defecation desire	997 (42.4)	4.6 (2.2–7.0)	<0.001	2.9 (0.7–5.2)	<0.01
Straining	960 (40.8)	7.9 (5.6–10.3)	<0.001	2.8 (0.5–5.2)	<0.05
Painful defecation	452 (19.2)	8.9 (6.0–11.9)	<0.001	1.7 (−1.2 to 4.6)	0.253
Infrequent defecation	1176 (50.0)	3.2 (0.9–5.5)	<0.01	−0.2 (−2.5 to 2.0)	0.857
Lumpy/hard stools	975 (41.5)	2.0 (−0.4 to 4.4)	0.098	−2.4 (−4.8 to −0.1)	<0.05

Effective number of patients was 2351.

CI, confidence interval.

### Total work productivity impairment or total activity impairment by presence or absence of each symptom

**Figure 4:**
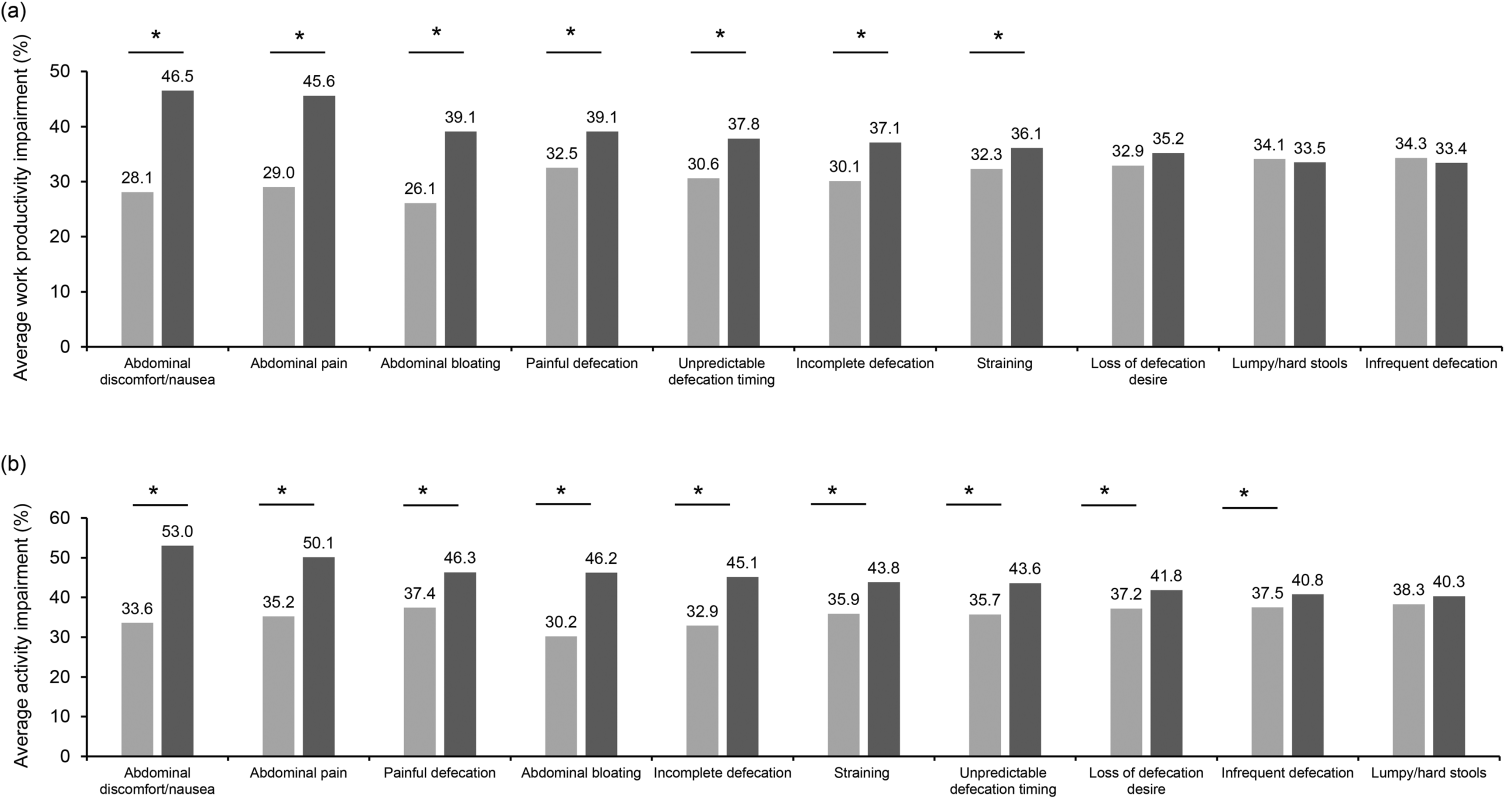
(a) Total work productivity impairment by presence or absence of each symptom. (b) Total activity impairment by presence or absence of each symptom. (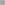), Without symptoms; (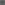), with symptoms. *Significance level *P* < 0.05, *t* test.

In the case of total work productivity impairment among the patients with and without constipation symptoms, statistically significant (*P* < 0.05) differences were found for symptoms such as abdominal discomfort/nausea, abdominal pain, abdominal bloating, painful defecation, straining, incomplete defecation, and unpredictable defecation timing. However, total activity impairment was significantly (*P* < 0.05) affected by all constipation symptoms except lumpy/hard stools (Fig. 4).

### Improvement in work productivity and daily activity after treatment

Laxative treatment improved work productivity and daily activity in 71.2% (95% CI: 68.6–73.6%) and 72.6% (95% CI: 70.8–74.4%) of patients, respectively.

## Discussion

Chronic constipation is a symptom‐based functional gastrointestinal disorder affecting people of all ages, both sexes, and different cultural and ethnic backgrounds. Though it is rarely life‐threatening, it has a significant negative impact on patients' QoL and consequently, productivity at work.^13^, ^17^, ^18^ Through this web‐based survey, we attempted to understand the correlation between different constipation symptoms and the degree of work productivity impairment. We found that abdominal symptoms and unpredictable defecation timing positively correlated with the loss of work productivity, and majority of patients noted an improvement in work productivity and daily activity with laxative use.

The patients of this survey were not balanced in terms of sex and age and included a high ratio of younger females. Some of them suffered from mental illness, and a substantial number were troubled by chronic constipation for 10 years or longer. A similar higher prevalence (about 67–80%) in females was also reported in previous studies.^11^, ^12^, ^13^ In a meta‐analysis on the prevalence of chronic idiopathic constipation, a higher rate in older adults compared to the younger population was observed,^19^ which is in contrast to our results where most patients were young females.

In our study, more than 70% of patients were prescribed magnesium oxide for the treatment of chronic constipation, which supports previously published reports showing that magnesium oxide is a widely used laxative in as many as 10 million patients in a year in Japan.^20^ The most common symptoms experienced by the patients in our study such as abdominal bloating, incomplete defecation, infrequent defecation, unpredictable timing of laxation, loss of defecation desire, lumpy/hard stools, and straining were similar to those reported in previous studies.^9^, ^21^ Symptoms experienced with the highest frequencies in previously published reports were straining and lumpy/hard stools.^9^, ^21^ On the other hand, the most frequent symptoms observed by 50% of patients in our study were infrequent defecation, abdominal bloating, and incomplete defecation. This difference in the proportion of symptoms might be due to laxative treatment‐induced improvement in symptoms in our study or geographical variations.

A previously published cross‐sectional, observational study in Japan has reported similar results to our study in terms of total work impairment (35.2% *vs* 33.9% in our study), activity impairment (36.8% *vs* 39.2% in our study), presenteeism (33.2% *vs* 31.2% in our study), and absenteeism (8.8% *vs* 5.0% in our study).^13^ The annual work productivity loss in terms of cost reported by Tomita *et al*. (1.218 million Japanese Yen) is also in agreement with our results (1.343 million Japanese Yen) with the slight difference being possibly attributable to differences in calculation method. Furthermore, by definition, IBS and chronic constipation are mutually exclusive, though opinions differ about this. Therefore, we analyzed the impact of chronic constipation symptoms on work productivity and daily activity impairment by excluding patients with IBS. However, the results did not change materially (data not shown). This estimated health burden is higher than that reported for patients with type 2 diabetes, IBS, and gastroesophageal reflux disease.^13^ It can be concluded that chronic constipation imposes a significant financial burden on patients despite being treated at hospitals with prescription laxatives.

Abdominal symptoms, such as abdominal bloating, abdominal pain, and abdominal discomfort/nausea, are known disease burdens for patients with chronic constipation. Heidelbaugh *et al*. reported that chronic idiopathic constipation patients with abdominal symptoms suffered more disrupted productivity than those without abdominal symptoms.^21^ This is consistent with our finding that abdominal symptoms were positively correlated with total work productivity impairment. In addition to work productivity, abdominal symptoms are reported to affect health‐related QoL. In a survey conducted among a random sample of American patients suffering from chronic constipation, more than half (52%) the patients reported their QoL being negatively impacted by abdominal symptoms.^9^ Similar trends have also been reported among Japanese patients.^22^ These observations suggest that abdominal symptoms are distressing and burdensome for patients with constipation.

In the present study, infrequent defecation and lumpy/hard stools did not positively correlate with work productivity and daily activity. These symptoms are included in the diagnostic Rome IV criteria for functional constipation and are often used as endpoints in clinical trials evaluating the efficacy of laxatives. Other symptoms included in the Rome IV criteria, such as residual stools and hardness, correlated with activity impairment but did not significantly correlate with work productivity. However, these symptoms affect QoL, indicating that the factors affecting QoL and work productivity may differ. Since all patients included in this study were prescribed laxatives, it is possible that the treatment with laxatives may have affected the observations.

While many constipation symptoms can be managed by laxatives, controlling the time of laxation remains a challenge.^23^ Previous studies have reported high rates of bowel urgency in patients with constipation, with unwanted timing of bowel urgency being associated with daily activity loss and anxiety.^24^ Unpredictable timing of laxation correlated with loss of work productivity and daily activity in our study. There are reports of high rates of bowel urgency experienced by constipated patients, and unwanted timing of bowel urgency is assumed to be more unproductive. In a survey involving 400 physician respondents and 700 patient respondents, it was observed that despite receiving treatment for chronic constipation, infrequent bowel movement was not sufficiently improved, leading to dissatisfaction among the patients^25^ and possibly impacting QoL and productivity. If defecation timing can be controlled by laxatives, it may be possible to achieve scheduled defecation by administering the doses at specific times, and loss of work productivity and daily activity can be minimized.

While the majority of patients (70%) in this study felt that their work productivity improved due to treatment, 30% did not feel satisfied, which remains a concern. Laxative use improved abdominal symptoms and increased the number of defecations without residual stools,^26^, ^27^, ^28^, ^29^ suggesting that the improvement of constipation symptoms reduced the loss of work productivity and daily activity. To further reduce this loss, it may be desirable to make treatment and laxative choices with attention to the unpredictable timing of laxation.

Limitations of this study included the lack of a control arm. Further, patients could have been excluded if they used over‐the‐counter medication for constipation control since the inclusion criteria restricted the enrollment to patients who visited a hospital and were treated with prescription laxatives. Since this was a web‐based survey, it can be assumed that the number of elderly patients was low because it is likely that fewer elderly people may have been inclined or comfortable to respond online. Only the presence or absence of improvement in work productivity and daily activity by laxatives was evaluated through a retrospective questionnaire, and therefore, recall bias may be assumed. Also, it is not clear which type of laxative can be expected to improve which symptoms and to what extent. Further, since the study enrolled patients already using laxatives for chronic constipation, we could not analyze whether their symptoms improved or not after drug treatment. Further, all included patients had at least one comorbid condition, and conditions such as gastroesophageal reflux disease might have contributed to abdominal symptoms potentially not responding to laxatives, thereby affecting study outcomes.

Symptoms of constipation, especially abdominal symptoms and unpredictable defecation timing, correlate with impaired work productivity and daily activity, affecting the patients' QoL and imposing a financial burden on them. Therefore, treatment focused on these symptoms may reduce the overall social and economic burden of constipation.

## Patient consent statement

Electronic informed consent was provided by all patients enrolled in the survey.

## Supporting information


**Table S1.** A questionnaire on health conditions (WPAI:CC questionnaire).

## Data Availability

Anonymized data not published within this article will be made available on request from any qualified investigators.
