# Supplementary material for: Impact of chronic constipation symptoms on work productivity and daily activity: A large‐scale internet survey
Source: JGH Open. 2024 Nov 4;8(11):e70042. doi: 10.1002/jgh3.70042 (PMC11532788; doi:10.1002/jgh3.70042)
Supplement: Supplementary file 1 — Table S1. A questionnaire on health conditions (WPAI:CC questionnaire). [file JGH3-8-e70042-s001.docx]

**A Questionnaire on Health Conditions**

Supplementary table 1 is**"A Questionnaire on Health Conditions (WPAI:CC questionnaire)".**

| **S1** | Please choose your gender. (Single answer)   1. Male 2. Female | | |  |
| --- | --- | --- | --- | --- |
| **S2** | How old are you?  _____ years old | | |  |
| **S3** | Which of the following do you fit into regarding your occupation? (Single answer)   1. I work in one of the following industries/fields: pharmaceutical/medical equipment, advertising/broadcasting, research/consulting. 2. I do not work in the above industries/fields. | | |  |
| **From here, we are asking about your own health conditions.** | | | |  |
| **S4** | Please choose from below all diseases and symptoms you have been diagnosed with by physicians.  Also, of the above, please choose all for which you are currently using medication prescribed from medical institutions.  (Multiple answers; as many as applicable)   \|  \| Diseases, symptoms you have been diagnosed with \| Of the ones chosen on the left, the ones for which you are currently using prescribed medication \| \| --- \| --- \| --- \| \| 1 Diabetes \| 1 \| 1 \| \| 2 Hypertension \| 2 \| 2 \| \| 3 Dyslipidemia \| 3 \| 3 \| \| 4 Gout, hyperuricemia \| 4 \| 4 \| \| 5 Chronic renal failure, uremia \| 5 \| 5 \| \| 6 Chronic constipation \| 6 \| 6 \| \| 7 Reflux esophagitis (GERD) \| 7 \| 7 \| \| 8 Irritable bowel syndrome (IBS) \| 8 \| 8 \| \| 9 Cerebrovascular disease \| 9 \| 9 \| \| 10 Parkinson’s disease \| 10 \| 10 \| \| 11 Mental illness (depression, schizophrenia, etc.) \| 11 \| 11 \| \| 12 None of the above \| 12 \| 12 \| | | |  |
| **From here, the questions are for people who are now using medication prescribed from medical institutions for "chronic constipation”** | | | |  |
| **S5** | Please choose from below all products that you are prescribed with for chronic constipation.  We recommend you to refer to your medication logbook (Okusuri-techo) if you have one.  (Multiple answers; as many as applicable)   1. Magnesium oxide (Magmitt, JuKama, heavy magnesium oxide) 2. Amitiza (Lubiprostone) 3. Goofice (elobixibat hydrate) 4. Linzess (linaclotide) 5. Movicol (polyethylene glycol combination) 6. Lagnos (lactulose) 7. Stimulant laxative (Sennoside, Pursennid, Alosenn, Picosulfate, Laxoberon, castor oil, etc.) 8. Chinese herbal medicine (Daio-Kanzo-To, Keishi-Kashaku-Yakuto, Mashi-Ningan, etc.) 9. Suppositories 10. Enema 11. Other laxatives (please describe: compulsory when selected) 12. Don't know; Don't remember | | |  |
| **Research on Labor Productivity Outcomes Related to Chronic Constipation** | | | |  |
| **S6** | May we ask you to cooperate in this research?   1. Yes, I agree to the above and am willing to cooperate with this research. 2. No, I am not willing to cooperate with this research. | | |  |
| **From here, we will be asking about the influence of chronic constipation on your work or other daily activities.** | | | |  |
| *In the following questions are those that ask you about how much time you missed from or put into your work.  Please respond with the total time during the past 7 days. (Not an average per day.) | | | |  |
| **Q1** | Are you currently employed (working for pay)? (Single answer)   1. NO 2. YES   **The next questions are about the past seven days, not including today.** | | |  |
| **Q2** | | During the past seven days, how many hours did you miss from work because of your chronic constipation?  Include hours you missed on sick days, times you went in late, left early, etc., because of your chronic constipation.  Do not include time you missed to participate in this study.  _______HOURS *Please input TOTAL time during the past 7 days. (Not the average time per day.) | |  |
| **Q3** | | | During the past seven days, how many hours did you miss from work because of reasons other than your chronic constipation: such as vacation, holidays, time off to participate in this study?  _______HOURS *Please input TOTAL time during the past 7 days. (Not the average time per day.) | |
| **Q4** | | | During the past seven days, how many hours did you actually work?  _______HOURS *Please input TOTAL time during the past 7 days. (Not the average time per day.) | |
| **Q5** | | | During the past seven days, how much did your chronic constipation affect your productivity while you were working?  Think about days you were limited in the amount or kind of work you could do, days you accomplished less than you would like, or days you could not do your work as carefully as usual.  If chronic constipation affected your work only a little, choose a low number.  Choose a high number if chronic constipation affected your work a great deal.  Consider only how much chronic constipation affected productivity while you were working. (Single answer)   \| Chronic constipation had no effect on my work \| 0 \| 1 \| 2 \| 3 \| 4 \| 5 \| 6 \| 7 \| 8 \| 9 \| 10 \| Chronic constipation completely prevented me from working \| \| --- \| --- \| --- \| --- \| --- \| --- \| --- \| --- \| --- \| --- \| --- \| --- \| --- \| | |
| **Q6** | | | **The next question is about the past seven days, not including today.**  During the past seven days, how much did your chronic constipation affect your ability to do your regular daily activities other than work at a job?  By regular activities, we mean the usual activities you do, such as work around the house, shopping, childcare, exercising, studying etc. Think about times you were limited in the amount or kind of activities you could do and times you accomplished less than you would like.  If chronic constipation affected your activities only a little, choose a low number.  Choose a high number if chronic constipation affected your activities a great deal.  Consider only how much chronic constipation affected your ability to do your regular daily activities, other than work at a job. (Single answer)   \| Chronic constipation had no effect on my daily activities \| 0 \| 1 \| 2 \| 3 \| 4 \| 5 \| 6 \| 7 \| 8 \| 9 \| 10 \| Chronic constipation completely prevented me from doing my daily activities \| \| --- \| --- \| --- \| --- \| --- \| --- \| --- \| --- \| --- \| --- \| --- \| --- \| --- \| | |
| **Q7** | | | **From here, we will be asking you about symptoms of your chronic constipation.**  Please choose all from below that fit the symptoms of your current chronic constipation. (Multiple answers; as many as applicable)   1. Don't feel the urge to defecate so much. 2. Unable to feel the timing of my stools. 3. Have pain in the abdomen. 4. Have discomfort in the abdomen. Feel nauseated. 5. Feel strained due to bloating in the abdomen. Abdomen feels heavy. 6. Painful when passing stools. 7. Passing fewer stools. 8. Have to strain (in the abdomen) to pass stools. 9. Have lumpy (like those of rabbit's stools) or hard (sausage-like form, but with uneven lumps) stools. 10. Feel like I haven't emptied the stool from the rectum. | |
| **Q8** | | | How long have you been having these symptoms of chronic constipation?  Please choose one from below that seems the closest. (Single answer)   1. Less than 5 years 2. Between 5 years to less than 10 years 3. 10 years or longer 4. Don't remember | |
| **Q9** | | | **From here are questions on treatment of chronic constipation.**  Please indicate how satisfied you are with the medication currently prescribed from medical institutions for your chronic constipation.  (Single answer)   \| Very satisfied \| Satisfied \| Somewhat satisfied \| Hard to say \| Not so satisfied \| Not satisfied \| Not satisfied at all \| \| --- \| --- \| --- \| --- \| --- \| --- \| --- \| | |
| **Q10** | | | Did the medication prescribed from medical institutions improve your work productivity?  Please think about how it was before being on drug therapy, to choose your response from below.  Think about days you were limited in the amount or kind of work you could do, and days  you accomplished less than you would like, or days you could not do your work as carefully as usual.  (Single answer)   1. It did improve. 2. It didn't improve. 3. I cannot compare things as I was not working before going on drug therapy. | |
| **Q11** | | | Did the medication prescribed from medical institutions improve your ability to do your regular daily activities  other than work? Please think about how it was before being on drug therapy, to choose your response from below  By regular activities, we mean the usual activities you do, such as work around the house, shopping, childcare, exercising, studying etc.  Think about times you were limited in the amount or kind of activities you could do and times you accomplished less than you would like.  (Single answer)   1. It did improve. 2. It didn't improve. | |
